# Supplementary material for: Expression of HOXB7 in the Lung of Patients with Idiopathic Pulmonary Fibrosis: A Proof-of-Concept Study
Source: Biomedicines. 2024 Jun 13;12(6):1321. doi: 10.3390/biomedicines12061321 (PMC11201217; doi:10.3390/biomedicines12061321)
Supplement: Supplementary file 1 [file biomedicines-12-01321-s001.zip › biomedicines-2993551-supplementary.pdf]

S1

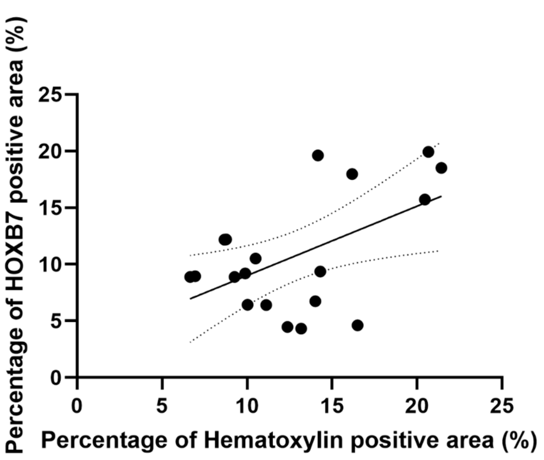

Figure S1. Simple linear regression between HOXB7 and hematoxylin expression of IPF patients as percentage of the positive area shown with significant positive correlation between them (r = 0.53 95% CI [0.116–1.114], p = 0.0158).

S2

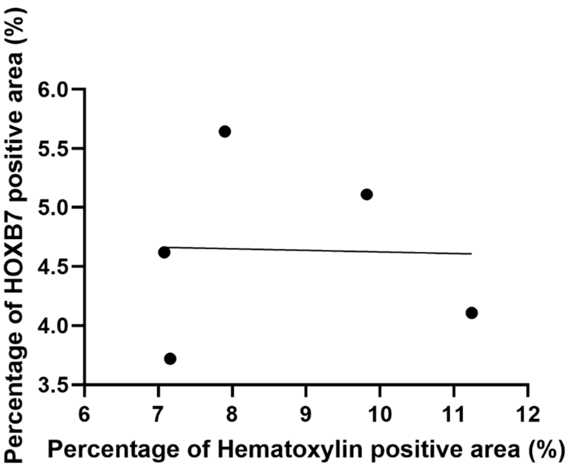

Figure S2. Simple linear regression between HOXB7 and hematoxylin expression of control patients as percentage of the positive area shown with non-significant positive correlation between them (r = <0.1 CI [−0.78–0.75], p = 0.96).

S3

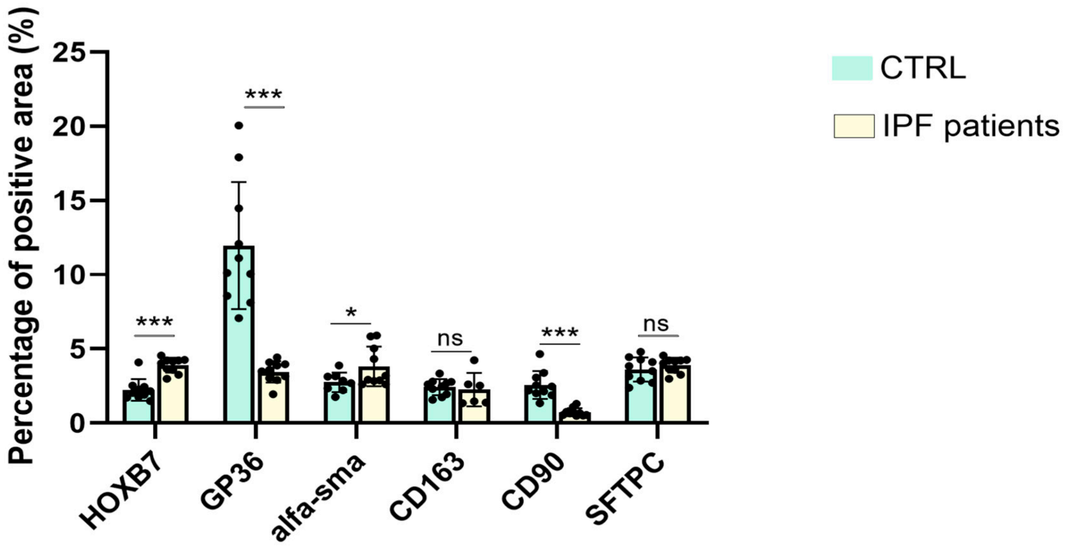

Figure S3. Unpaired parametric *t*-test, two-tailed between CTRL and IPF patients showing significant difference in the positive area for α-SMA staining (* p-value = 0.048, difference between means 1.162 ± 0.518), CD90 (*** p-value < 0.001, difference between means 1.837 ± 0.307), GP-36 (*** p-value < 0.001, difference between means −8.5 ± 1.37), and HOXB7 (*** p-value < 0.001, difference between means 1.66 ± 0.3279) while differences in the positive area for CD163 (p-value = 0.384, difference between means −0.169 ± 0.41) and SFTPC (p-value = 0.369, difference between means 0.279 ± 0.303) was not significant. Error bars represent the standard error of the mean difference.

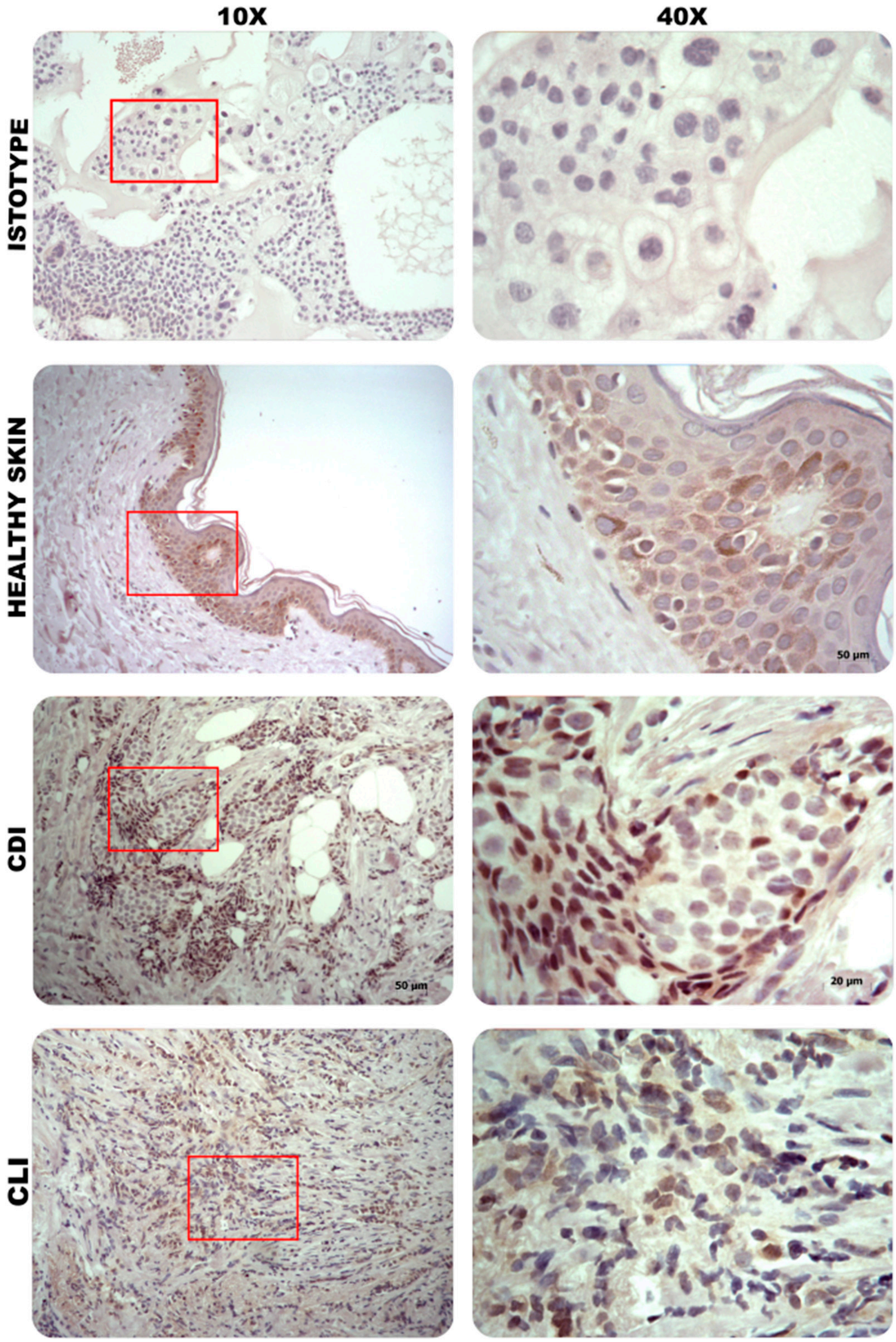

Figure S4. Photomicrograph showing immunohistochemistry (5 µm thick) of isotype as negative control with 10× and 40× magnifications, scale bar 50 µm. Photomicrographs showing immunohistochemistry (5 µm thick) of isotype as negative control with 10× and 40× magnifications, scale bar 50 µm. Photomicrographs showing immunohistochemistry with HOXB7 antibody on healthy skin tissue (5 µm thick) as positive control with 10× and 40× magnifications, scale bar 50 µm. Photomicrographs showing immunohistochemistry with HOXB7 antibody on invasive ductal carcinoma tissue (5 µm thick) as positive control with 10× and 40× magnifications, scale bar 20 µm. Photomicrographs showing immunohistochemistry with HOXB7 antibody on invasive lobular carcinoma tissue (5 µm thick) as positive control with 10× and 40× magnifications, scale bar 20 µm.
